# Geographic disparities in late stage breast cancer incidence: results from eight states in the United States

**DOI:** 10.1186/s12942-015-0025-5

**Published:** 2015-10-24

**Authors:** Zaria Tatalovich, Li Zhu, Alicia Rolin, Denise R. Lewis, Linda C. Harlan, Deborah M. Winn

**Affiliations:** Division of Cancer Control and Population Sciences, National Cancer Institute, National Institutes of Health, Bethesda, MD 20892 USA; Surveillance Research Program, Division of Cancer Control and Population Sciences, National Cancer Institute, 9609 Medical Center Dr. Suite 4E 446, Rockville, MD 20850 USA

**Keywords:** Cancer, Late stage, Geographic disparities, Breast cancer

## Abstract

**Background:**

Late stage of cancer at diagnosis is an important predictor of cancer mortality. In many areas worldwide, cancer registry systems, available data and mapping technologies can provide information about late stage cancer by geographical regions, offering valuable opportunities to identify areas where further investigation and interventions are needed. The current study examined geographical variation in late stage breast cancer incidence across eight states in the United States with the objective to identify areas that might benefit from targeted interventions.

**Methods:**

Data from the Surveillance Epidemiology and End Results Program on late stage breast cancer incidence was used as dependent variable in regression analysis and certain factors known to contribute to high rates of late stage cancer (socioeconomic characteristics, health insurance characteristics, and the availability and utilization of cancer screening) as covariates. Geographic information systems were used to map and highlight areas that have any combination of high late stage breast cancer incidence and significantly associated risk factors.

**Results:**

The differences in mean rates of late stage breast cancer between eight states considered in this analysis are statistically significant. Factors that have statistically negative association with late stage breast cancer incidence across the eight states include: density of mammography facilities, percent population with Bachelor’s degree and English literacy while percent black population has statistically significant positive association with late stage breast cancer incidence.

**Conclusions:**

This study describes geographic disparities in late stage breast cancer incidence and identifies areas that might benefit from targeted interventions. The results suggest that in the eight US states examined, higher rates of late stage breast cancer are more common in areas with predominantly black population, where English literacy, percentage of population with college degree and screening availability are low. The approach described in this work may be utilized both within and outside US, wherever cancer registry systems and technologies offer the same opportunity to identify places where further investigation and interventions for reducing cancer burden are needed.

## Background

Breast cancer is the top cancer in women worldwide and is increasing particularly in developing countries where the majority of cases are diagnosed in late stages [1]. Late stage of cancer at diagnosis is an important predictor of cancer mortality. Understanding geographic disparities in late stage of breast cancer at diagnosis is critical for cancer control activities. In many areas worldwide, cancer registry systems, available data and mapping technologies can provide information about late stage cancer by geographic regions, offering valuable opportunities to identify places where further investigation and interventions are needed. The objective of present study is to demonstrate the use of available data and geographic information systems to (1) examine geographic disparities in late stage breast cancer incidence, (2) identify factors that are associated with higher rates of late stage breast cancer across different geographic areas, and (3) highlight areas that might benefit from targeted interventions. It is hoped that the approach offered in this work will be utilized broadly and outside of US, where cancer registry systems and technologies offer the same opportunity to identify places that require specific cancer control interventions to reduce cancer burden.

### Late stage breast cancer (LSBC): theoretical foundation

Many factors contribute to LSBC at diagnosis. Among the major factors are the underlying biological aggressiveness of the disease [2]; demographic and socio-economic characteristics [3–6], health insurance status [7, 8], accessibility to healthcare and diagnostic services [9–11], the availability and utilization screening tests [12].

Studies suggest that black women are more often diagnosed with LSBC than white women [13–15]. Furthermore, African-American/Black women are more likely than other US race and ethnic groups to develop aggressive breast cancer that is estrogen and progesterone receptor negative for negative for human epidermal growth factor and with distant metastases at diagnosis [16]. Evidence of ethnic disparities suggests that Hispanic women experienced lower incidence rates of LSBC than non-Hispanic women [17].

Financial resources have been associated with breast cancer stage. Low socioeconomic status and poverty have been correlated with higher rates of late-stage breast cancer [5, 18, 19]. Other research evidence suggests that poor literacy limits patients’ understanding of cancer screening and of symptoms of cancer, potentially adversely affecting their stage at diagnosis [20].

In studies that examined health insurance, having no insurance or Medicaid coverage is associated with a higher proportion of LSBC [8, 21]. Kuzmiak [8] found that, compared to insured patients, uninsured patients had a 66 % higher likelihood of presenting with LSBC.

Furthermore, it has been suggested that accessibility to health care, measured by lower availability of mammography facilities, and/or primary care physicians—delays the detection of early stage breast cancer [10, 22–25].

Other evidence suggest that a lower density of mammography facilities has been associated with the higher proportion of late stage breast cancer at diagnoses in rural areas [9] where there is more limited access to mammography facilities [25] and women are less likely to have received a mammogram [26]. Conversely, some studies found that geographical access to mammography facilities and primary care providers were not correlated with stage at diagnosis [27]. McLafferty [28, 29] found an “urban disadvantage”—higher proportions of late stage breast cancer in urban areas.

Many of the aforementioned contributors to LSBC are correlated with each other, change over time, and vary across geographical areas [13, 18, 20, 21]. Some factors such as the availability, cost, and accessibility of mammography screening and diagnostic services are potentially modifiable and addressing them could result in more women being diagnosed earlier when the disease is more amenable to treatment.

The aforementioned evidence of the major contributors to LSBC served as the theoretical foundation for selection of particular variables in our study. The next section provides an overview of these variables and data sources.

## Methods

### Data and variables

Data from the Surveillance, Epidemiology, and End Results (SEER) Program of the United States National Cancer Institute [30] were used as a source of outcome (dependent) variable in the analysis—late stage breast cancer incidence. SEER is comprised of 18 population-based cancer registries covering 28 % of total US population. The information collected includes primary tumor site, tumor morphology and stage at diagnosis, first course of treatment, and follow-up for patient’s vital status. SEER provides baseline measures of cancer incidence rates, survival and prevalence statistics, and change in incidence and survival trends over time for several geographic units, listed in order from the smallest to the largest: County, Health Service Area, State, and Nation, and makes public use files available to support population-based cancer research.

This study utilized SEER data on patients who were diagnosed with LSBC in the following eight states that comprise 26 % of the US population: California, Georgia, Iowa, Louisiana, Kentucky, New Jersey, New Mexico, and Utah. Cases of LSBC were defined as female, age 40 years or older, diagnosed with LSBC between 2006 and 2010. Late stage was defined as stage III and stage IV, using the guidelines from AJCC (American Joint Commission on Cancer) 6th Edition Cancer Staging Handbook [31].

In this study, the outcome variable is age-adjusted incidence rates of late stage breast cancer (LSBC) for females, 40 years of age and older, that were diagnosed in the period between 2006 and 2010 (the rates were age-adjusted to 19 age groups using the 2000 US standard population).

The independent variables in the analysis represent some of the major factors that were found to contribute to LSBC including: socio-demographic and economic characteristics (race, ethnicity, literacy, education, median income, health insurance coverage); accessibility to health care (urban–rural residence); availability of screening services (density of obstetrics and gynecology physicians and FDA approved mammography facilities) and utilization of mammography (percentage of women who utilized mammogram). The variables tested in the model with a detailed definition, data sources, and data timeline are shown in Table 1.

**Table 1:** Variables and data sources

Variables	Definition	Data sources
Dependent variables		
Late stage breast cancer diagnosis ages 40 and above	Age-adjusted incidence rates of late stage breast cancer in females, ages 40 and above, per 100,000 women diagnosed from 2006 to 2010	SEER http://www.seer.cancer.gov/data
Predictor variables		
Density of mammography facilities per person	Number of FDA approved mammography facilities in 2010, normalized by the total population in 2010	FDA http://www.accessdata.fda.gov/scripts/cdrh/cfdocs/cfmqsa/mqsa.cfm
% Screening	Percent of females, ages 40 and above that had mammography in the past 2 years; estimates for the period 2000–2003	Small Area Estimates (SAE) sae.cancer.gov
Density of obstetrics and gynecology specialists per person	Number of OBGYN specialists in 2010, normalized by the total population in 2010	HRSA Area Resource File (ARF) http://arf.hrsa.gov/
% Urban households	Percent urban households (including urbanized areas and clusters) in 2010	US Census Bureau2010 http://www.census.gov/2010census/
% Black	Percent of total population that is Black in 2010	US Census Bureau 2010 http://www.census.gov/2010census/
Median household income	Median household Income in 2010	US Census Bureau 2010 http://www.census.gov/2010census/
% English litreracy	Percentage of population 5 years and older that speaks English “very well” in 2010	US Census Bureau 2010 http://www.census.gov/2010census/
% With college degree	Percent of total population with a Bachelor’s degree or higher in 2010	US Census Bureau 2010 http://www.census.gov/2010census/
% With private insurance	Percent of female population, ages 40+ with private insurance only in 2010	US Census Bureau 2010 http://www.census.gov/2010census/
% Uninsured	Percent of female population, ages 40+ with no health insurance coverage in 2010	US Census Bureau 2010 http://www.census.gov/2010census/

The socio-demographic data were obtained from the US Census Bureau 2010 data file, and include the following variables: percentage of Black population; median household income, English literacy, education, health insurance coverage, and rural–urban residence.

Data on the number of OBGYN specialists in defined geographic areas was obtained from the Health Resources and Services Administration (HRSA) area resources file (ARF) [34]—estimates for the year 2010. These data were normalized by the total population to calculate a measure of OBGYN specialists per person.

The number of FDA approved mammography facilities was available from the US Food and Drug Administration (FDA)—estimates for the year 2010. These data were normalized by the total population in order to generate density measure of the number of facilities per person.

Breast cancer screening measures were available from the small area estimates (SAE) file [33]. These are model-based, bias-corrected measures of the percentage of female population ages 40 and older who reported having a mammography test in the past 2 years—estimates for the period 2000–2003.

### Geographic unit of analysis

Data for all variables in this analysis were initially assigned to a county, the largest administrative unit in the United States. However, since the LSBC data are too sparse to provide stable 5-year incidence rates at the county level, the data were aggregated into health service areas (HSA’s) in order to assure stability of rates. HSA’s were originally defined by the National Center for Health Statistics as larger geographic areas comprised of one or more counties and are defined such that most residents in the region obtain hospital care from the same set of hospitals [32]. The original HSAs were modified by The National Cancer Institute so that any HSA that crossed state or SEER Registry boundaries were split and all counties from one HSA were in one state and/or SEER Registry. There are 944 HSAs in the US that contain 3141 counties according to the modified HSA definition. Since the modified HSAs are delineated using geopolitical boundaries of counties and states, this makes them compatible with many data systems (e.g. census data), thus increasing the possibilities for data analysis [32].

### Analysis

All statistical analyses were performed in SPSS 16.0 software. We plotted the mean rates of late stage breast cancer by SEER state with confidence intervals; subsequently we ran Analysis of Variance (ANOVA) with Bonferroni adjustment to test whether or not the observed variations in the mean rates of LSBC were statistically significant between the states (p < 0.05), and if so, which states differ significantly. Secondly, we classified the HSA-level incidence rates of LSBC for all eight SEER states using tertiles and computed the proportion of HSAs in each class by state. In addition we generated a map of incidence rates of LSBC to show the location of HSAs with high, medium, or low rates across the eight SEER states. In the next step, we ran the “backward” stepwise linear regression to determine the factors that best explain LSBC incidence for females, ages 40 and older. Our analysis included the ten potential predictor variables listed in Table 1 and individual states as covariates. Backward stepwise regression essentially does multiple regression a number of times, each time removing the weakest correlated variable. The final model contains variables that best explain the variation in the dependent variable. Finally, we mapped the strongest predictors of LSBC by HSA of each SEER state in order to examine their geographic distributions and identify areas that may be in need of intervention. All maps were created using ESRI (Environmental Systems Research Institute, Inc. Redlands, CA) ArcGIS 10.1 software.

## Results

**Fig. 1:**
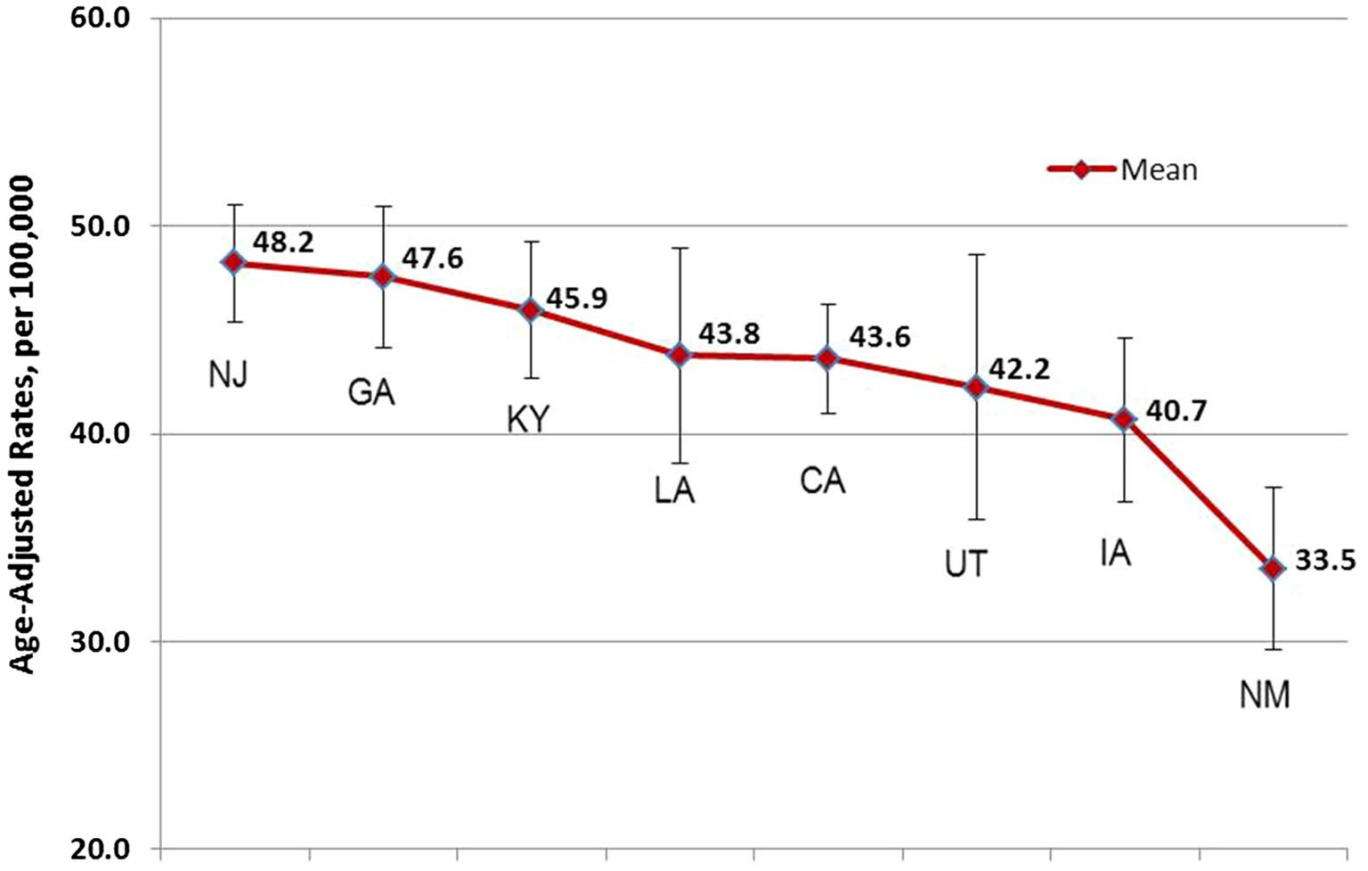
Mean rates and confidence intervals of LSBC incidence, females, Ages 40 and above, 2006–2010

**Fig. 2:**
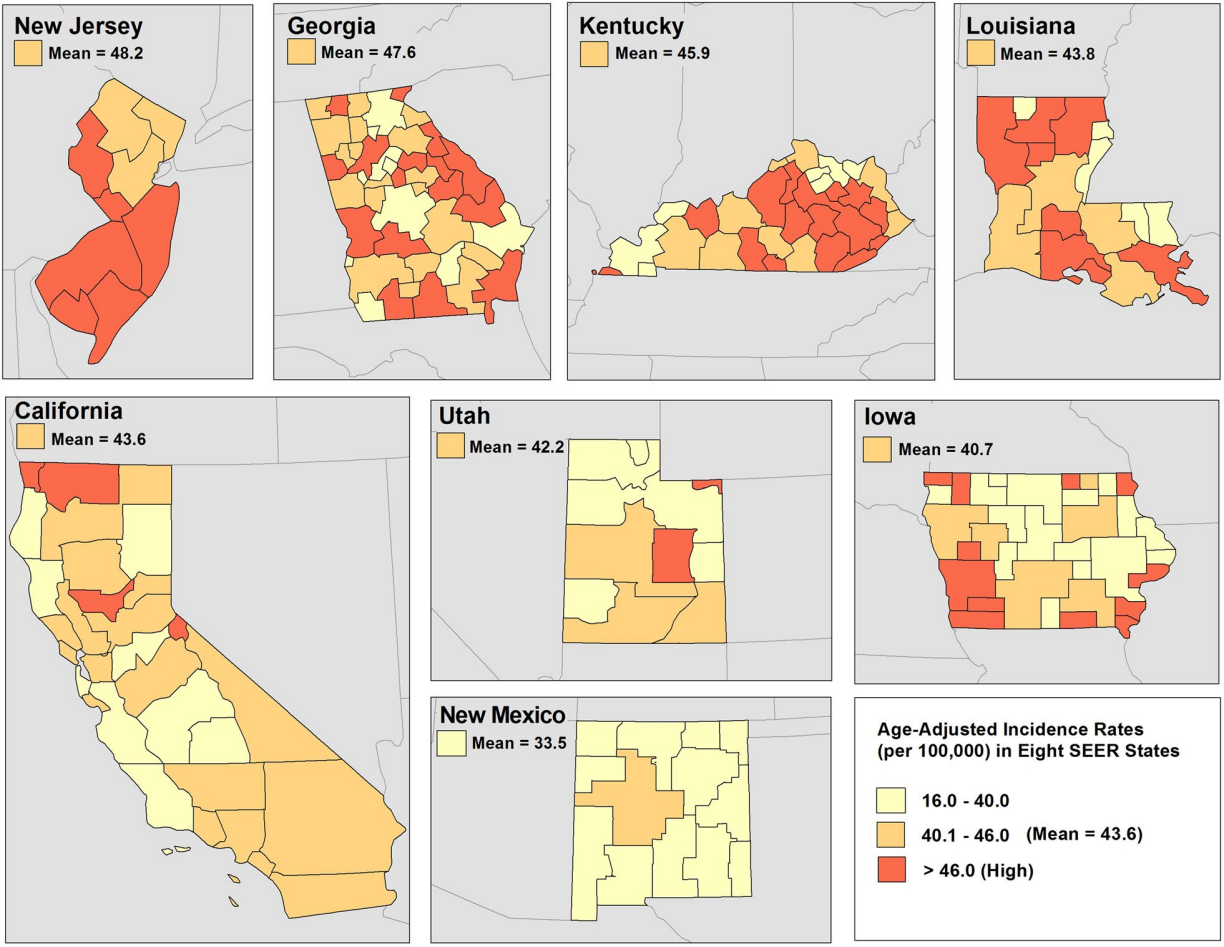
LSBC incidence by HSA, females, ages 40 and above, 2006–2010

Figures 1 and 2 illustrates the variation in the mean rates of LSBC for women 40 years of age and older, by state. The mean for eight SEER states is 46.3 per 100,000 women. Ranked in order from highest to lowest state, New Jersey has, on an average, the highest incidence rates of LSBC (48.2 per 100,000 women), followed by Georgia, Kentucky, Louisiana, California, Utah, Iowa, and New Mexico with the lowest (33.6 per 100,000).

To test for significant differences in late stage breast cancer incidence among the eight states, we run an analysis of variance and get an F statistic value of 4.241, with p < 0.0001, which indicates that the variation in mean rates of LSBC by state is statistically significant. The results of Bonferoni test (Table 2) reveal where the significant differences lie: New Mexico has, on an average, significantly lower incidence rates of LSBC than New Jersey (p < 0.0001), Georgia (p < 0.0001), Kentucky (p < 0.0001), and California (p < 0.003). There are no significant differences in mean rates of LSBC between other states.

**Table 2:** States that have significantly different LSBC incidence rates

	(I) State Mean	(J) State Mean	Mean difference (I − J)	Std. error	Sig.	95 % confidence interval	
						Lower bound	Upper bound
Late stage breast cancer incidence, females ages 40 and above	NM (33.5)	NJ (48.2)	−14.7*	2.1781	0.000	−22.057	−7.327
		GA (47.6)	−14.1*	2.4642	0.000	−21.996	−6.107
		KY (45.9)	112.4*	2.4045	0.000	−20.245	−4.623
		CA (43.6)	−10.1*	2.2174	0.003	−17.420	−2.778

No differences between mean rates for Utah and Iowa

* Statistically significant difference

Figure 2 represents a map of LSBC incidence by HSA. Incidence rates are classified into tertiles (low, medium, and high values) and percent contribution of HSAs to each class of values is summarized in Table 3: the state with the highest mean (48.2 per 100,000 women)—New Jersey, has 70 % of HSAs with high incidence rates of LSBC (>46.0 per 100,000 women), and the remaining 30 % with medium rates (40.1–46.0). In contrast, New Mexico—the state with the lowest mean (33.5) has 80 % of HSAs with low rates (16.0–40.0) and 20 % with medium rates. In California, most of the HSAs (60 %) have medium rates. Georgia, Kentucky, and Louisiana each have greater proportion of HSAs in the medium and high range, respectively, while Utah and Iowa have proportionately more HSAs in low and medium ranges.

**Table 3:** Percentage of HSAs with low, medium, or high rates of LSBC by SEER state

State	16.0–40.0 (low) (%)	40.1–46.0 (med) (%)	>46.0 (high) (%)
New Jersey	0	30	70
Georgia	20	40	40
Kentucky	20	30	50
Louisiana	30	20	50
California	30	60	20
Utah	40	40	20
Iowa	45	20	25
New Mexico	80	20	0

Table 4 shows the descriptive statistics for variables that were entered into a stepwise regression, while Table 5 reveals which of those variables best explain the variation in LSBC incidence. The hypothesis being tested is: there is no association between late stage breast cancer and each of the predictors, assuming other predictors are associated with LSBC. We rejected the null hypothesis based on the following results: model R = 0.493 and adjusted R^2^: 0.205, indicating that about 20 % of the total variance in LSBC incidence rates is explained by the regression model. The standardized coefficient shows that mammography density is the most strongly associated predictor of LSBC with a negative effect. On average, for each unit increase in Mammography density, the LSBC incidence rate decreases by 0.383.

**Table 4:** Descriptive statistics for variables entered in the regression analysis

Variables	Mean	Range
Late stage breast cancer diagnosis ages 40 and above—dependent variable	43.81	62.90
Density of mammography facilities per person	3.99	14.22
% Screening	0.66	0.32
Density of obstetrics and gynecology specialists per person	7.70	25.44
% Urban households	0.56	1.00
% Black	0.12	0.61
Median household income	46205	68985
% English literacy	5.14	26.68
% With college degree	0.10	0.41
% With private insurance	0.49	0.45
% Uninsured	0.11	0.40

States	Number of HSAs per State
NM, Reference	13
CA	30
GA	42
IA	34
KY	31
LA	17
NJ	9
UT	10

**Table 5:** Best model fit: effects of state, demographic, and health characteristics on LSBC incidence rates

HSA characteristics	Unstandardized coefficients		Standardized coefficients	t	Sig.
	B	Std. error	Beta		
(Constant)	48.871	2.970		16.456	0.000
Density of mammography facilities per person	−0.541	0.156	−0.383	−3.457	0.001
% With college degree	−36.433	13.964	−0.243	−2.609	0.010
% Black	13.879	4.993	0.239	2.779	0.006
% English literacy	−0.836	0.406	−0.174	−2.058	0.041
New Jersey	10.069	2.786	0.289	3.614	0.000
California	9.105	2.320	0.428	3.924	0.000
Kentucky	4.039	1.984	0.179	2.035	0.044

Other HSA-level factors that explain the variation in LSBC across the eight SEER States include: percent of population with college degree or higher, English literacy (both significantly negatively associated with LSBC incidence), and percent of Black population (significantly positively associated with LSBC). After considering these four predictors, the states of New Jersey, California, and Kentucky still have significantly higher rates of LSBC than New Mexico (the reference category).

**Fig. 3:**
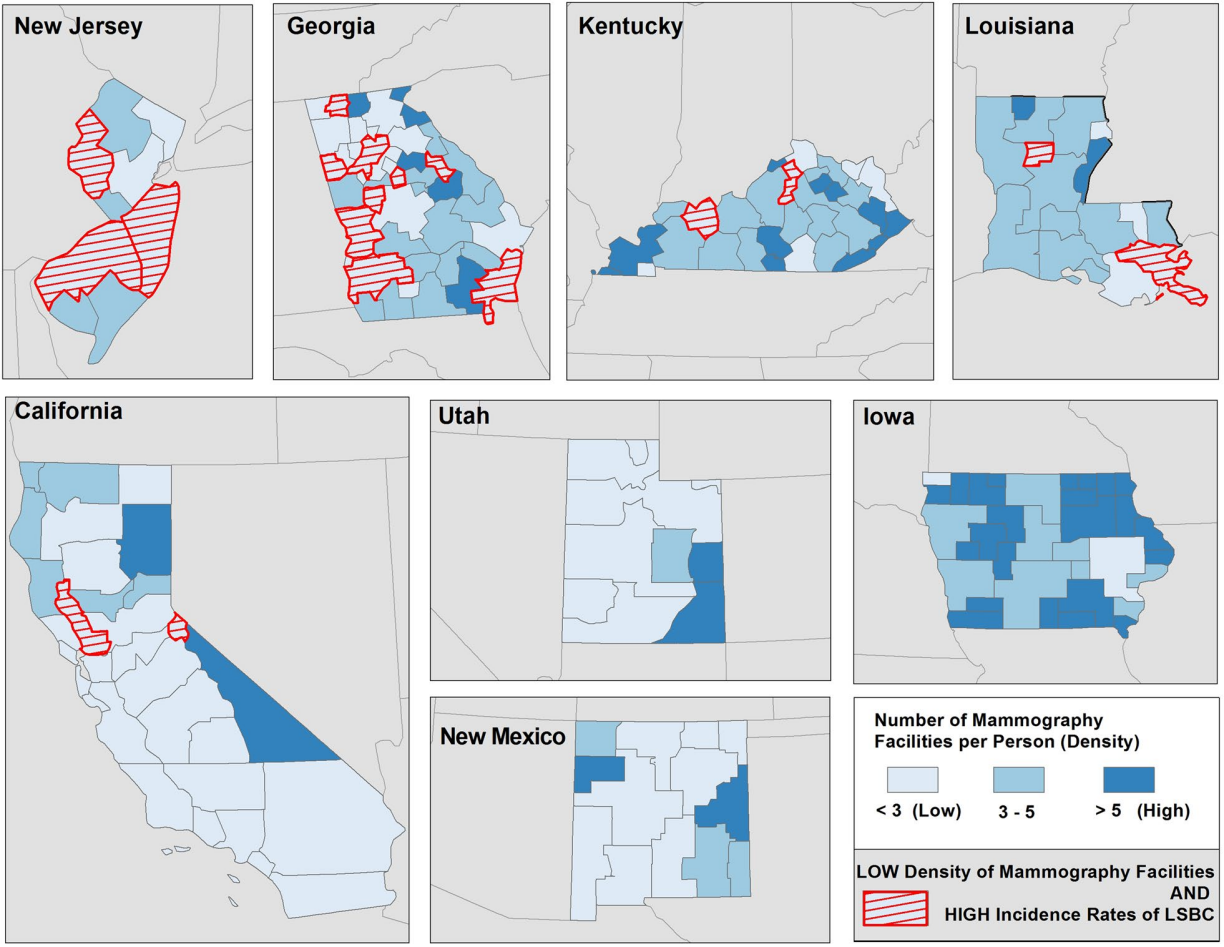
Number of FDA approved mammography facilities per person by HSA

**Fig. 4:**
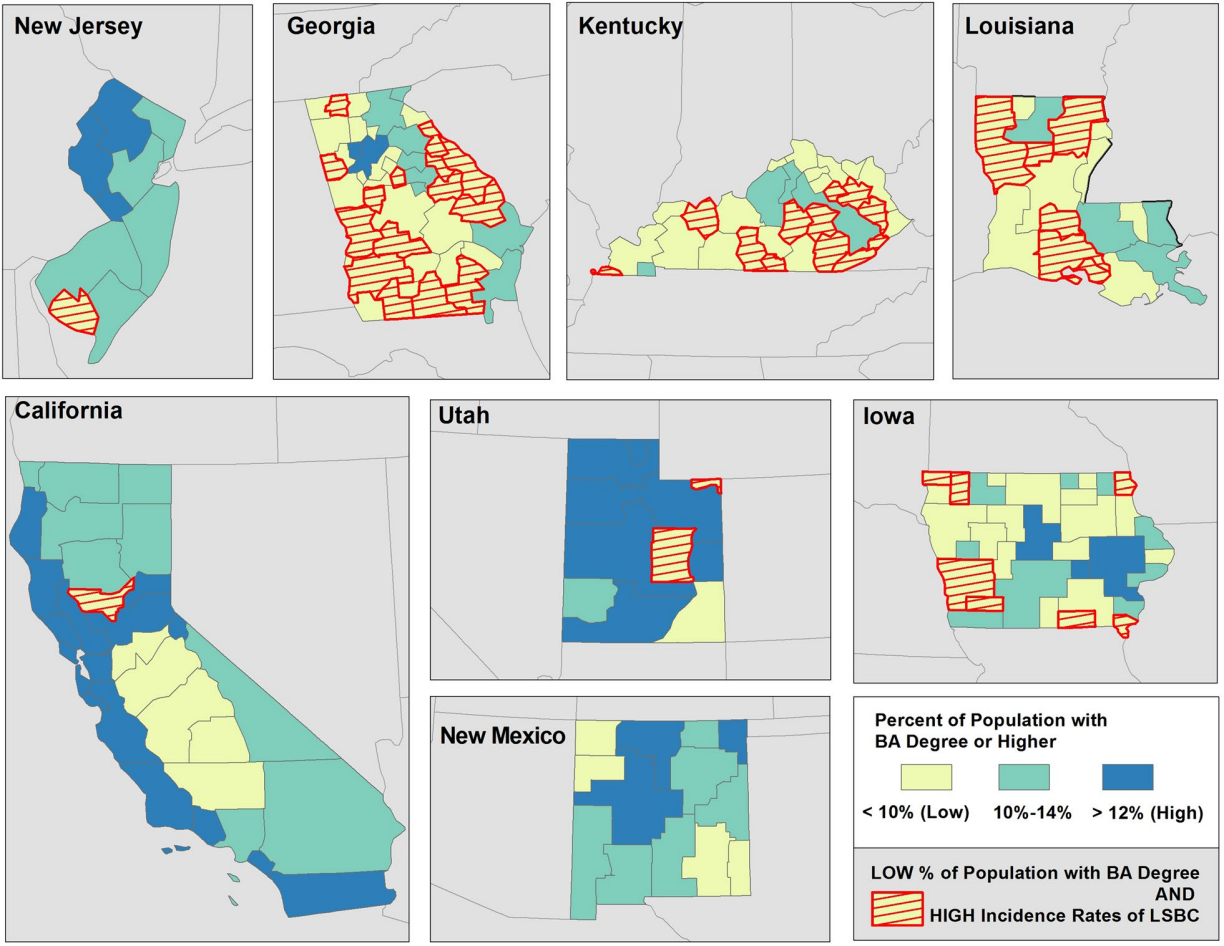
Percent of population with BA degree or higher by HSA

**Fig. 5:**
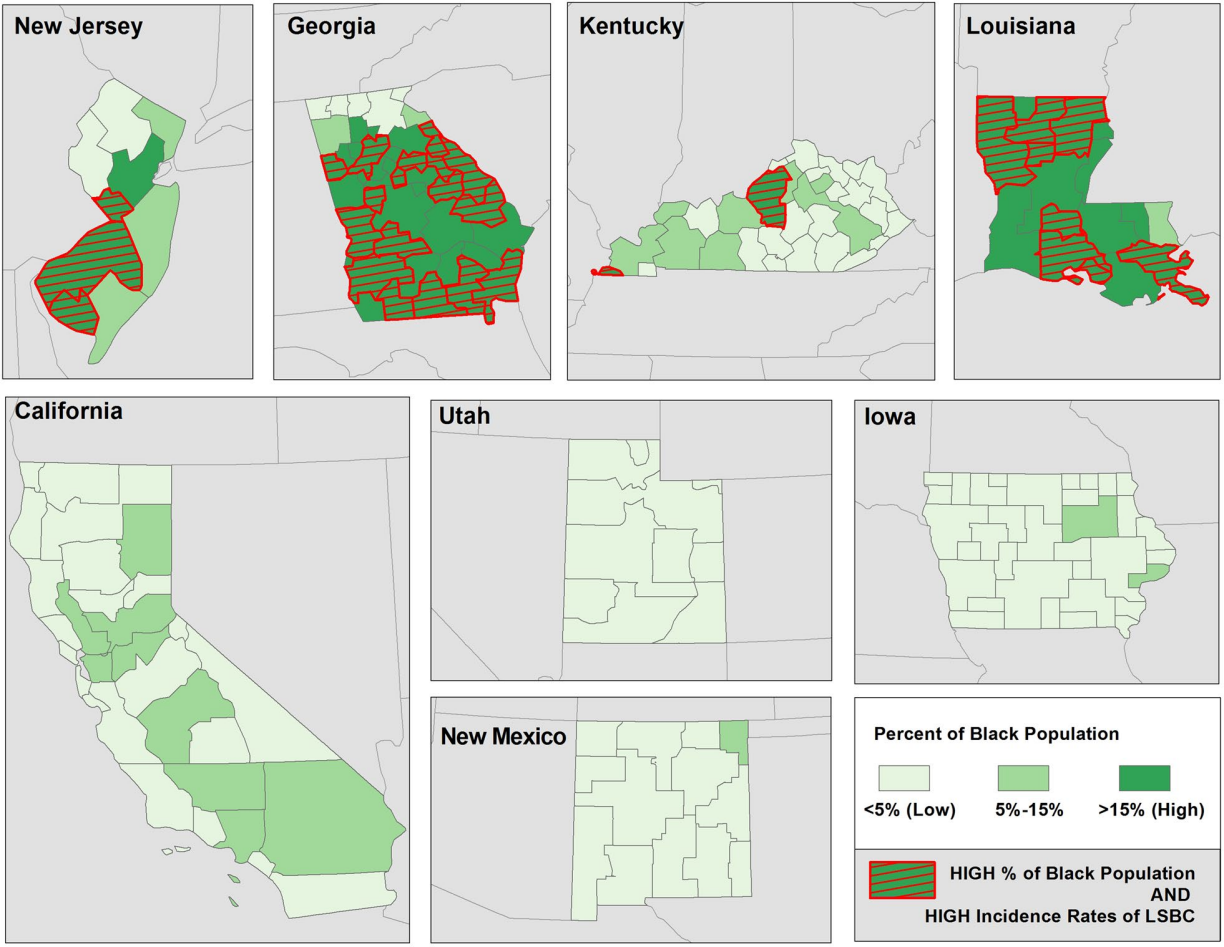
Percent of black population by HSA

The maps illustrate geographic disparities in: Density of FDA approved mammography facilities (Fig. 3), population with BA degree or higher (Fig. 4) and Black population (Fig. 5)—the three strongest predictors of LSBC found in this study. More importantly, the maps highlight *where* to focus targeted interventions. Our model suggests that areas with low density of mammography, low educational attainment, and high percentage of Black population tend to have higher incidence rates of late stage breast cancer. In the first map (Fig. 3), red-shaded areas represent HSAs with high incidence rates of LSBC (>46.0 per 100,000) and, at the same time, with less than 3 FDA approved mammography facilities per person. In the second map (Fig. 4), shaded areas highlight HSAs with high incidence rates of LSBC and, at the same time, relatively low educational attainment (less than 10 % of population having BA degree or higher). The last map (Fig. 5) highlights the areas where both the incidence of LSBC and proportion of Black population are high.

## Conclusions

In this study we sought to describe the geographic disparities in late stage breast cancer incidence across eight states in the US and identify areas where LSBC are common, and where further research could help better identify reasons for the high incidence of late stage diagnoses and interventions could be used to modify factors contributing to the high rates of LSBC. For example, identification of areas with higher rates of LSBC and factors contributing to them may help identify where resources might be needed to increase screening for breast cancer and provide greater availability of services that can provide more aggressive treatments.

We found heterogeneity across the eight states examined in the incidence of late stage diagnosis, with the state with the highest percent, New Jersey at 48 per 100,000 women, having an incidence rate 30 % higher than that of the lowest, New Mexico, where the rate was 33.5 per 100,000 women. Our results indicate that New Mexico has significantly lower incidence rates of LSBC than four other states.

We also found that lower density of screening mammography units within HSAs were associated with higher rates of late stage breast cancer in our sample. This is consistent with prior findings regarding lack of accessibility to health care—whether measured by lower availability of mammography facilities and primary care physicians—delays the detection of early stage breast cancer [10, 11, 22–25]. Other studies suggest that a lower density of mammography facilities units has been associated with the higher proportion of late stage breast cancer at diagnoses in rural areas [9] where there is more limited access to mammography facilities [26] and women are less likely to have received a mammogram [27]. Our population-based sample covered all parts of the states studied, and so included both urban areas as well as rural areas. In our study, however, the percentage of the HSA’s population that was urban did not contribute to late stage breast cancer rates.

McLafferty [28, 29] found an “urban disadvantage”—higher proportions of late stage breast cancer in urban areas. The driving factor of late stage breast cancer may be limited use of mammography facilities whether the barrier is access and distance or demographic and socioeconomically based. Again, our study does not support nor contradict these findings, given that the urban–rural factor was not significant predictor of late stage.

Financial resources have been associated with breast cancer stage. For example, low socioeconomic status and poverty have been correlated with higher rates of late-stage breast cancer [5, 18, 19]. In the current study the percentage of people within an HSA who had a college education was inversely associated with rates of late stage breast cancer across the eight states. A college education is likely a surrogate for a higher income as well as higher educational attainment. In addition, it has been suggested that poor literacy limits patients’ understanding of cancer screening and of symptoms of cancer, potentially adversely affecting their stage at diagnosis [20]. In our study, poor English literacy was significantly associated with higher rates of late stage breast cancer.

In studies that examined health insurance, having no insurance or Medicaid coverage is associated with a higher proportion of LSBC [8, 21]. In the current study an increased percentage of people within HSAs who had private health insurance did not contribute to late stage breast cancer rates.

The US cancer registry systems and available data and mapping technology can provide detailed information about late stage breast cancer by geographical regions, offering valuable opportunities to identify cancer-related health disparities and areas where further investigation and interventions are needed. We mapped and highlighted Health Service Areas that have any combination of high late stage breast cancer incidence and significantly associated factors; the obvious motivation was to identify areas that might benefit from targeted interventions.

Further work, however is needed in order to capture additional factors that drive the differences in LSBC incidence among states, and that did not come as significant in our model. Identifying underlying reasons for geographic variation presents many challenges, including missing data and measurement issues. In addition, some area level data is collected and released for research in varying time periods; this is a limitation of our study where there is a substantial variation in timeline of some data; this could have affected our results if there were secular trends over time for specific predictors or LSBC. In addition, factors available at the geographical level are often imprecise or do not fully capture important underlying domains. For example, the density of obstetricians and gynecologists, used as an indicator of one type of physician who commonly refers women for mammographic screening; or the density of mammographic facilities as a proxy for the capacity of the geographic area to provide mammograms to the population of women are inherently limited. Nevertheless reducing cancer-related geographic disparities is an important goal.

Strengths of the study included population-based cancer registries that capture almost 100 % of all cancers in defined geographic areas, well-documented and standardized methods across states for categorizing data from registries into stage, coverage of roughly 28 % of the US population, and the opportunity to compare late stage rates across states in contrast to most earlier research studies, which focused on smaller geographic units. Geo-spatial analysis of patterns of late stage breast cancer can be useful to inform targeted interventions in the areas that need them the most.

In conclusion, this study suggests that in the eight US states examined, higher rates of late stage breast cancer are more common in areas with predominantly black population (disparity in race), where English literacy, percentage of population with college degree (disparity in socio-demographic characteristics) and screening availability are low. The approach described in this work may be utilized both within and outside US, wherever cancer registry systems and area-level potential predictor variables and mapping technologies are available to identify and better characterize areas with high risks of cancer or cancer-related outcomes that may benefit from further investigation and interventions to reduce the cancer burden.

## Abbreviations

LSBC: late stage breast cancer
SEER: Surveillance, Epidemiology, and End Results Program
AJCC: American Joint Commission on Cancer
HAS: health service area
FDA: Food and Drug Administration
SAE: small area estimates
BRFSS: the behavioral risk factor surveillance system
NHIS: National Health Interview Survey
OBGYN: obstetrics and gynecology
HRSA: Health Resources and Services Administration
ARF: area resources file
ANOVA: analysis of variance
ESRI: Environmental Systems Research Institute

## References

[1] Global Health Estimates. WHO 2013. http://www.who.int/cancer/detection/breastcancer/en/index1.html.

[2] Verma R, Bowen RL, Slater SE, Mihaimeed F, Johnes JL (2012). Pathological and epidemiological factors associated with advanced stage at diagnosis of breast cancer. Br Med Bull.

[3] Gerend M, Pai M (2008). Social determinants of black-white disparities in breast cancer mortality: a review. Cancer Epidemiol Biomarkers Prev..

[4] Campbell RT, Li X, Dolecek TA, Barrett RE, Weaver KE, Warnecke RB (2009). Economic, racial and ethnic disparities in breast cancer in the US: towards a more comprehensive model. Health Place.

[5] Tian N, Wilson JG, Zhan FB (2011). Spatial association of racial/ethnic disparities between late-stage diagnosis and mortality for female breast cancer: where to intervene?. Int J Health Geogr.

[6] MacKinnon JA, Duncan RC, Huang Y, Lee DJ, Fleming LE, Voti L, Rudolph M, Wilkinson JD (2007). Detecting an association between socioeconomic status and late stage breast cancer using spatial analysis and area-based measures. Cancer Epidemiol Biomarkers Prev.

[7] Roetzheim RG, Ferrante JM, Lee JH, Chen R, Love-Jackson KM, Gonzales EC, Fisher KJ, McCarthy EP (2012). Influence of primary care on breast cancer outcomes among Medicare beneficiaries. Ann Fam Med..

[8] Kuzmiak CM, Haberle S, Padungchaichote W, Zeng D, Cole E, Pisano ED (2008). Insurance status and the severity of breast cancer at the time of diagnosis. Academic radiology.

[9] Onitilo AA, Liang H, Stankowski RV, Engel JM, Broton M, Doi SA, Miskowiak DA (2014). Geographical and seasonal barriers to mammography services and breast cancer stage at diagnosis. Rural Remote Health.

[10] Dai D (2010). Black residential segregation, disparities in spatial access to health care facilities, and late-stage breast cancer diagnosis in metropolitan Detroit. Health Place.

[11] Coughlin SS, Richardson LC, Orelien J, Thompson T, Richards TB, Sabatino SA, Wu W, Cooney D (2009). Contextual analysis of breast cancer stage at diagnosis among women in the United States, 2004. Open Health Serv Policy J.

[12] Onitilo AA, Engel JM, Liang H, Stankowski RV, Miskowiak DA, Broton M, Doi SA (2013). Mammography utilization: patient characteristics and breast cancer stage at diagnosis. Am J Roentgenol..

[13] Keller D, Guilfoyle C, Seriego J (2011). Geographical influence on racial disparity in breast cancer presentation in the United States. Am Surg.

[14] Clarke CA, Keegan THM, Yang J, Press DJ, Kurian AW, Patel AH, Lacey JV (2012). Age-specific incidences of breast cancer subtypes: understanding the black-white crossover. JNCI.

[15] Markossian TW, Hines RB (2012). Disparities in late stage diagnosis treatment, and breast cancer-related death by race, age, and rural residence among women in Georgia. Women Health.

[16] Kohler BA et al. Annual Report to the Nation on the Status of Cancer, 1975–2011, featuring incidence of breast cancer subtypes by race/ethnicity, poverty, and state. J Natl Cancer Inst. 2015. 10.1093/jnci/djv048 PMC4603551 25825511

[17] Clegg LX, Reichman ME, Miller BA, Hankey BF, Singh GK, Lin YD, Goodman MT, Lynch CF, Schwartz SM, Chen VW, Bernstein L, Gomez SL, Graff JJ, Lin CC, Johnson NJ, Edwards BK (2009). Impact of socioeconomic status on cancer incidence and stage at diagnosis: selected findings from the surveillance, epidemiology, and end results: National Longitudinal Mortality Study. Cancer Causes Control.

[18] Barry J, Breen N, Barrett M (2012). Significance of increasing poverty levels for determining late-stage breast cancer diagnosis in 1990 and 2000. J Urban Health.

[19] Henry K, Sherman R, Farber S, Cockburn M, Goldberg D, Stroup A (2013). The joint effects of census tract poverty and geographic access on late-stage breast cancer diagnosis in 10 US States. Health Place.

[20] Davis CT, Williams VM, Marin E, Parker MR, Glass J (2002). Health literacy and cancer communication. CA Cancer J Clin.

[21] Freedman RA, Virgo KS, He Y, Pavluck AL, Winer EP, Ward EM, Keating NL (2010). The association of race/ethnicity, insurance status, and socioeconomic factors with breast cancer care. Cancer.

[22] Wang F, McLafferty S, Escamilla V, Luo L (2008). Late-stage breast cancer diagnosis and health care access in Illinois. Prof Geogr.

[23] Lian M, Struthers J, Schootman M (2012). Comparing GIS-based measures in access to mammography and their validity in predicting neighborhood risk of late-stage breast cancer. PLoS One.

[24] Mobley LR, Kuo TM, Watson L, Gordon Brown G (2012). Geographic disparities in late-stage cancer diagnosis: Multilevel factors and spatial interactions. Health Place.

[25] Jones AP, Haynes R, Sauerzapf V, Crawford SM, Zhao H, Forman D (2008). Travel time to hospital and treatment for breast, colon, rectum, lung, ovary and prostate cancer. Eur J Cancer.

[26] Elkin EB, Ishill NM, Snow JG, Panageas KS, Bach PB, Liberman L, Wang F, Schrag D (2010). Geographic access and the use of screening mammography. Med Care.

[27] Doescher MP, Jackson JE (2009). Trends in cervical and breast cancer screening practices among women in rural and urban areas of the United States. J Public Health Manag Pract.

[28] McLafferty S, Wang F, Luo L, Butler J (2011). Rural–urban inequalities in late-stage breast cancer: spatial and social dimensions of risk and access. Environ Plan.

[29] McLafferty S, Wang F (2009). Rural–Urban disparities in late stage breast cancer risk in Illinois. Cancer.

[30] Surveillance Epidemiolgy and End Results (SEER) Program, N.C.I. Health Service Areas (HSA). 2010. http://seer.cancer.gov/seerstat/variables/countyattribs/hsa.html. Accessed Jan 2015.

[31] Greene FL, Page DL, Fleming ID et al. (eds). AJCC Cancer Staging Manual, 6th edition. 2002; p. 435.

[32] Makuc DM (1991). Health service areas for the United States. Vital Health Stat.

[33] National Cancer Institute. Small area estimates for cancer risk factors and screening behaviors. 2014. http://sae.cancer.gov/. Accessed Jan 2015.

[34] Health Resources and Services Administration. Area Resource File (ARF), National County-level Health Resource Information Database. 2011. http://www.arf.hrsa.gov/. Accessed Jan 2015.

